# Efficient Compressive Strength Prediction of Alkali-Activated Waste Materials Using Machine Learning

**DOI:** 10.3390/ma17133141

**Published:** 2024-06-27

**Authors:** Chien-Hua Hsu, Hao-Yu Chan, Ming-Hui Chang, Chiung-Fang Liu, Tzu-Yu Liu, Kuo-Chuang Chiu

**Affiliations:** Material and Chemical Research Laboratories, Industrial Technology Research Institute, Hsinchu 31040, Taiwan; haoyuchan@itri.org.tw (H.-Y.C.); mirandachang@itri.org.tw (M.-H.C.); liucf@itri.org.tw (C.-F.L.); jill.t.y.liu@itri.org.tw (T.-Y.L.); ckc@itri.org.tw (K.-C.C.)

**Keywords:** alkali-activated materials (AAMs), optimization, component classification, compressive strength, machine learning

## Abstract

This study explores the integration of machine learning (ML) techniques to predict and optimize the compressive strength of alkali-activated materials (AAMs) sourced from four industrial waste streams: blast furnace slag, fly ash, reducing slag, and waste glass. Aimed at mitigating the labor-intensive trial-and-error method in AAM formulation, ML models can predict the compressive strength and then streamline the mixture compositions. By leveraging a dataset of only 42 samples, the Random Forest (RF) model underwent fivefold cross-validation to ensure reliability. Despite challenges posed by the limited datasets, meticulous data processing steps facilitated the identification of pivotal features that influence compressive strength. Substantial enhancement in predicting compressive strength was achieved with the RF model, improving the model accuracy from 0.05 to 0.62. Experimental validation further confirmed the ML model’s efficacy, as the formulations ultimately achieved the desired strength threshold, with a significant 59.65% improvement over the initial experiments. Additionally, the fact that the recommended formulations using ML methods only required about 5 min underscores the transformative potential of ML in reshaping AAM design paradigms and expediting the development process.

## 1. Introduction

Alkali-activated materials (AAMs) are increasingly acknowledged for their environmentally friendly characteristics and reduced carbon footprint [1]. A key factor in fostering the principles of the circular economy is the utilization of alkali activation technology. Alkali-activated materials (AAMs), encompassing geopolymers, are of interest due to their ability to utilize industrial by-products and waste materials, thereby further enhancing their environmental benefits. Thapa et al. [2] propose that the reaction mechanisms of AAM and geopolymer differ. AAMs, containing higher calcium content in their raw materials, form a binder gel after dissolution in alkaline solutions of moderate to high alkalinity, resulting in phases of calcium silicate hydrate/aluminate hydrate and an alumino-silicate network structure. In contrast, geopolymers, with lower calcium content in their raw materials, form a polymeric structure after dissolution in alkaline solutions of lower alkalinity, ultimately resulting in an alumino-silicate network structure [3,4,5]. AAMs exhibit wide-ranging applications across various industrial sectors, bolstering their commercial viability [6]. Manufacturing AAMs using industrial waste materials, such as blast furnace slag [7,8], fly ashes [9,10], metallurgical slags [11,12], or waste glass [13,14,15], presents two benefits: they not only involve the utilization of waste products to create value-added products, but also significantly decrease the environmental impact across many industries by conserving vast amounts of raw materials [16,17].

However, the application of AAMs is currently confronting persistent engineering difficulties, such as loss of workability [18], rheological issues [19], cost concerns [20], the need for special types of curing [21], and the limited reactivity of precursors, especially for those derived from waste streams [22]. This necessitates the incorporation of alkaline activators, resulting in intricate chemical interactions. Additionally, the diverse range of raw materials poses challenges in formulating standardized mix designs. Despite these issues, optimism about the use of AAMs persists due to several compelling reasons.

Studies have demonstrated the excellent performance of AAMs and their remarkable effectiveness in various specialized engineering applications, such as their use as repair materials [23], in marine constructions [24], as pavement base materials [25], as 3D printing raw materials [26], and in the containment of hazardous waste materials [27]. The integration of environmentally conscious substitutes like AAMs in these specialized domains offers the potential for enhanced performance and longevity, contributing to the sustainability and resource efficiency of infrastructure. In 2023, research conducted by Liu et al. [28] using borax overcame the problem of AAMs being unable to solidify at room temperature, thereby avoiding the complexity and cost of thermal solidification. By solving these drawbacks, the practical applicability of AAMs in construction can be significantly enhanced, making them a more viable and sustainable alternative. Therefore, the belief is that future solutions to these engineering problems will not impede the feasibility of applying AAMs.

Conventional approaches to AAM design heavily rely on empirical experiments conducted by experienced operators [29,30]. These methods involve trial-and-error processes, demanding substantial time and resource investments [31,32]. The recent advancements in AI have significantly transformed materials science and computational design. By harnessing machine learning (ML) algorithms, researchers can now predict material properties, optimize mixtures, and expedite the discovery of novel materials. This AI-driven approach allows scientists to simulate and analyze various material compositions, dramatically reducing the need for extensive physical experiments and addressing the traditionally time-consuming validation cycles.

The assessment of compressive strength (CS), a crucial parameter in AAM design, typically involves 28 days of destructive compressive strength testing, even by experienced operators. While non-destructive methods exist for estimating compressive strength, direct and precise testing of compressive strength still requires materials to undergo destruction, making destructive compressive strength tests the mainstream analysis method. Additionally, the prolonged durations of trial-and-error experiments and curing periods consume substantial manpower and time [33,34,35,36]. Therefore, there is a critical need to develop rapid and effective mixture design methods to achieve optimal performance of AAMs.

ML has recently emerged as a powerful technique to predict the compressive strength of concrete using various algorithms [37,38,39,40,41,42,43,44]. Table 1 summarizes the findings from prior research relating to ML algorithms employed in predicting the diverse properties of concrete. Based on this literature, it is understood that at least 100 sets of concrete material data are required to establish a model robust enough for application. This is due to the significant variability in the composition of waste materials, necessitating a substantial amount of data for the potential development of applicable ML models. Thus, the size of a dataset plays a pivotal role in achieving high efficacy in terms of ML models, particularly in the field of materials science. Datasets in this field can vary widely, ranging from small-scale to extensive (e.g., small-scale (<100) to extensive (>1000)), and significantly impact the training processes of ML models [45]. In cases where the available data are insufficient, the resulting model might exhibit diminished predictive accuracy or struggle with generalization, leading to overfitting [46,47,48]. Therefore, it is highly desired to devise effective strategies to alleviate the adverse effects that arise from working with small datasets.

In this study, the model’s performance was validated using various evaluation metrics commonly used in the literature [49,50,51] to ensure its reliability. To our knowledge, this study is the first to apply ML techniques to investigate over 20 factors related to blast furnace slag (BFS), fly ash (FA), reducing slag (RS), and waste glass (WG) in the context of AAMs. The insights gained can guide the design of future BFS–FA–RS–WG-based AAMs and support the use of industrial waste materials in construction.

The main objective of this research is to develop a robust ML model for predicting the compressive strength of AAMs, addressing the challenges posed by the variability in raw material compositions and the limitations of small datasets. This study is original in its application of ML to AAMs, particularly in dealing with diverse recycled materials and small dataset constraints, which have not been extensively explored in previous research.

To address these challenges, this research achieved the following aims:

1. Establish ML models using experimental data from AAMs to accelerate the development of AAMs;
2. Overcome the challenges in building ML models with small datasets using data-processing methods;
3. Utilize the established ML models in conjunction with experimental validation to identify AAM formulations with high compressive strength.

## 2. Materials and Methods

### 2.1. Experimental Design and Dataset

(1) Raw materials

The dataset, provided by the Material and Chemical Research Laboratories (MCL) and Industrial Technology Research Laboratories (ITRI), is referred to as the “ITRI AAM database [52]”. It includes 42 samples, each characterized by various raw materials of Blast furnace slag (BFS), Fly ash (FA), Reducing slag (RS), Waste glass (WG).

(2) Physical and chemical characteristics

- The recipes for the AAMs consist of precursor activators (PAs), alkali activators (AAs), and process parameters such as mixing time (t) and alkali solution temperature (T).
- The precursor activators include BFS obtained from a steel plant (Taiwan), with a D_50_ of 9.5 µm, and fly ashes (FAs) obtained from three different power plants (Taiwan), with D_50_ values ranging from 14 μm to 15 μm.
- The alkali activators include RS obtained from three different steel plants (Taiwan), where the activator purities range from 0.071% to 0.050%, and the molar ratios of Na/Si range from 0.068% to 0.043%. The D_50_ of the RS powder ranges from 14 μm to 15 μm. Additionally, the alkali activators include WG obtained from four different glass plants (Taiwan), where the activator purities range from 2.180% to 6.959%, and the molar ratios of Na/Si range from 2.435% to 9.540%. The D_50_ of the WG powder ranges from 6 μm to 7 μm.
- For the alkali solutions, there are two types of alkali solutions (NaOH and Na_2_SO_4_), with 99% purity.
- The composition transformation dataset was obtained via an X-ray fluorescence analysis of the materials used. An automated spectrometer (RIX 200, manufactured by Thermo Fisher Scientific, Wilmington, MA, USA) was used to determine the chemical compositions. The key constituents include Fe_2_O_3_, Al_2_O_3_, SiO_2_, K_2_O, Na_2_O, CaO, and MgO. The minor constituents include ZrO_2_, B_2_O_3_, TiO_2_, Bi_2_O_3_, and SrO. The chemical composition analysis of the powder is shown in Table 2.

(3) Sample preparation and testing

The dataset includes experimental results relating to high-strength AAMs designed for construction applications. The samples were prepared by the ITRI–MCL laboratory as follows:

- Step 1: The alkali activators were mixed for 3 min and then cooled to room temperature;
- Step 2: The precursor activators were then stirred and mixed with the alkali activators for 10 min;
- Step 3: The paste was cast into molds and cured at room temperature;
- Step 4: The compressive strength was assessed using a 50 mm cube following ASTM C109 procedures [53], with three independent tests.

The process parameters concern the alkali solution mixing time (t): t1 for the alkali activators, and t2 for both the precursor activators (PAs) and alkali activators (AAs).

(4) Validation and application of ML models

The validation method involved several steps:

- Step 1: Experimental Validation
  The recommended recipes were tested experimentally. The results were compared to the predicted values, with the goal of achieving a target compressive strength of 30 MPa.
- Step 2: Model Improvement
  Additional experimental data were collected, expanding the dataset to 45 data points. The model was retrained, and new recipes were tested to assess prediction accuracy.
- Step 3: Final Validation
  The dataset was further expanded to 48 data points through continuous experimentation. New recipes were tested to confirm whether a target compressive strength of 30 MPa was consistently achieved.

### 2.2. Data Processing

**Figure 1:**
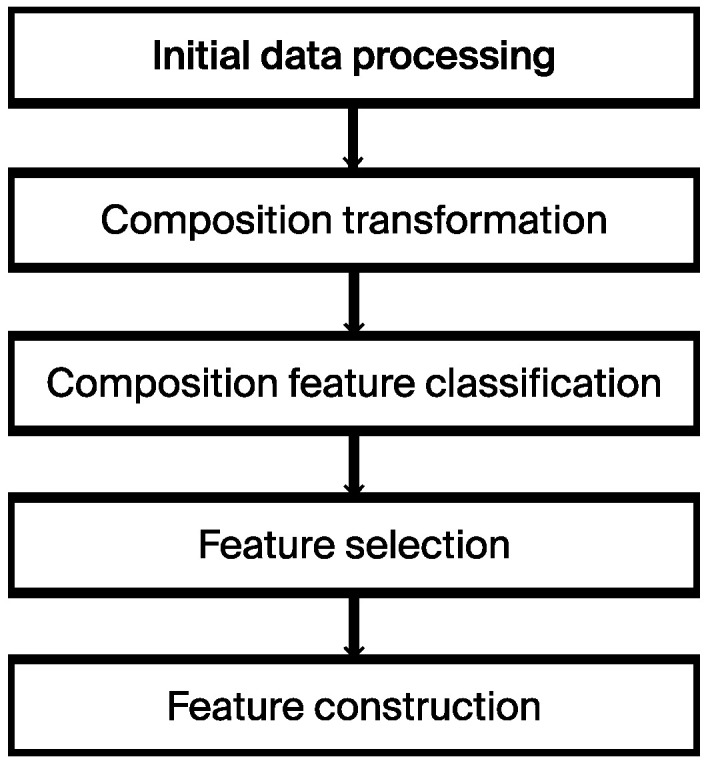
Flowchart of the proposed approach for data processing.

Data processing encompassed several stages: initial data processing, composition transformation, composition feature classification, feature selection, and feature construction. Figure 1 illustrates the overall process. The steps are defined as follows:

Initial data processing involved data cleaning and the preparation of the initial dataset. This process included identifying and handling missing values, addressing outliers, and performing data scaling and normalization to ensure data quality and consistency.

Composition transformation involves converting the composition data in the dataset into a format that is understandable by ML models.

At this stage, the composition data were classified based on their features for further analysis and modeling.

Feature selection chooses the most informative features from the dataset to improve the performance and effectiveness of the ML models.

Feature construction involved combining, transforming, and/or generating new features to enhance the performance and generalization of models.

This comprehensive approach aims to ensure data quality, enhance model performance, and facilitate subsequent analysis and modeling tasks.

#### 2.2.1. Composition Transformation

The composition transformation dataset was obtained via an X-ray fluorescence analysis of the materials used. An automated spectrometer (RIX 200, manufactured by Thermo Fisher Scientific, Wilmington, MA, USA) was used to determine the chemical compositions. Then, the individual components were combined in a dataset. Table S1 presents the statistical characteristics and distribution of the variables under investigation, providing the featured information of the composition transformation dataset containing 42 pieces of data. During the measurement, the process parameters and the target features remain unchanged, as indicated in Table 3.

#### 2.2.2. Composition Feature Classification

The composition feature classification dataset was derived from the composition transformation dataset. This process categorizes the precursor activator and alkali activator into separate columns based on their reaction mechanisms, as shown in Table 4. Given that the reaction mechanisms of precursor activator and alkali activator differ in actual chemical reactions, separating the compositions of these two distinct AAM mechanisms enables the ML model to effectively distinguish the impact of these materials on the compressive strength of AAMs. This separation aids in improving the model’s accuracy in predicting compressive strength. Table 4 presents the statistical characteristics and distribution of the investigated variables, providing the feature information of the composition feature classification dataset for these 42 pieces of data. During the measurement, the process parameters and target features remain unchanged, as indicated in Table 3.

#### 2.2.3. Feature Selection

The feature selection dataset removes composition fields with lower content, preserving only the fields of primary composition. The increased number of columns in the dataset after classification (adding an additional 14 columns) may lead to poor accuracy in ML models. Therefore, essential constituents affecting compressive strength were selected based on alkali activation principles [48]. These key constituents included Fe_2_O_3_, Al_2_O_3_, SiO_2_, K_2_O, Na_2_O, CaO, and MgO, along with a category denoted as “Others”. The “Others” category includes the remaining components, such as ZrO_2_, B_2_O_3_, TiO_2_, Bi_2_O_3_, and SrO, which are those other than the above-mentioned seven major constituents. Table S2 presents the statistical characteristics and distribution of the variables under investigation, providing the feature information of the feature selection dataset containing 42 pieces of data. During the measurement, the process parameters and target features remain unchanged, as indicated in Table 3.

#### 2.2.4. Feature Construction

The feature construction dataset includes the creation of new feature columns, such as volume molar concentration, based on alkali activation principles. In this study, a total of 6 features were established, all of which were calculated from the dataset filtered through key constituents of compressive strength. Table S3 presents the statistical characteristics and distribution of the variables under investigation in our study, providing the featured information of the feature construction dataset containing 42 pieces of data. During the measurement, the composition, process parameters, and target features remained unchanged, as indicated in Table 3 and Table S2.

### 2.3. Model Assessment

In this study, all of the data in the initial dataset were trained. To mitigate potential issues with poor model generalization, the model was evaluated using 5-fold cross-validation and assessed for performance using metrics such as Mean Absolute Error (MAE), Root Mean Squared Error (RMSE), and Coefficient of Determination (*R*^2^) values.

#### 2.3.1. Fivefold Cross-Validation

**Figure 2:**
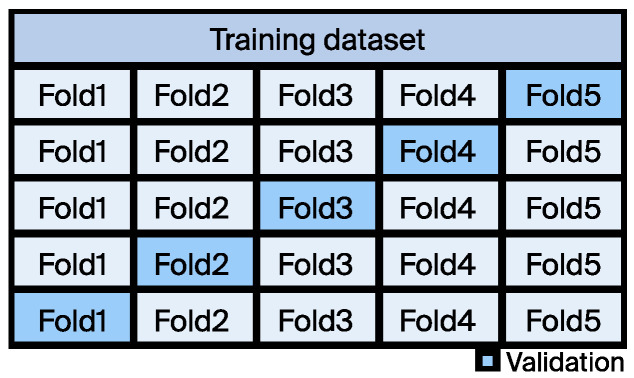
Schematic structure for the 5-fold CV.

Fivefold cross-validation was employed to assess the performance of the model, as shown in Figure 2. This method involves dividing the dataset into 5 subsets, using 4 subsets for training the model each time, and reserving the remaining subset for testing. This process was repeated 5 times, and each time used a different subset for testing. The final performance evaluation is the average of the results from the 5 tests.

#### 2.3.2. Error Matrices

##### Mean Absolute Error (MAE)

Mean Absolute Error (MAE) is a measure of the average difference between the predicted and the actual values.

The mathematical formula for MAE is as follows:

$$MAE=\frac{\sum_{i}^{n}\left|y-f\right|}{n}$$

The vector $y=(y_{1},\;y_{2},\cdots ,y_{n})$ represents the actual values of the target variable. The vector $f=(f_{1},\;f_{2},\cdots ,f_{n})$ represents the predicted values of the target variable.

##### Root Mean Squared Error (RMSE)

Root Mean Squared Error (RMSE) is a measure of the square root of the average of the squared differences between the predicted and the actual values. It provides a measure of the typical deviation of the predictions from the actual values.

The mathematical formula for RMSE is as follows:

$$RMSE=\sqrt{\frac{\sum_{i}^{n}{\left(y-f\right)}^{2}}{n}}$$

The vectors y and f are as above.

##### Coefficient of Determination (*R*^2^)

The mathematical formula for *R*^2^ is as follows:

$$R^{2}=1-\frac{\sum_{i}^{n}{\left(y-\bar{y}\right)}^{2}}{\sum_{i}^{n}{\left(y-f\right)}^{2}}$$

The vectors y and f are as above.

## 3. Results and Discussion

### 3.1. Input and Output Variables

**Figure 3:**
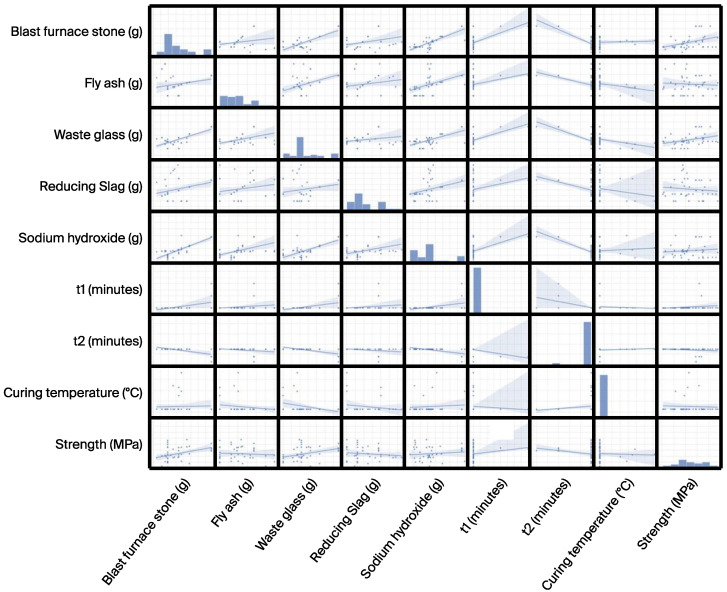
Pair plot of the initial dataset.

**Figure 4:**
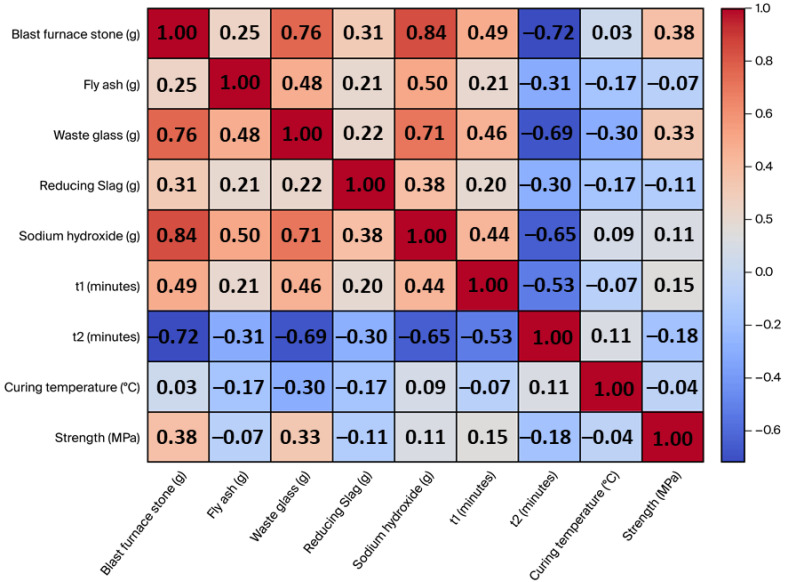
Pearson correlation matrix of the initial dataset.

Figure 3 illustrates a pair plot depicting the distribution of precursor activator, alkali activator, process parameters, and compressive strength (target feature). The pair plot is created using the Seaborn package [54]. The shaded areas in the plot result from setting the parameter “kind” to “reg” in the “pairplot” function. This means that a regression line is added to the scatter plots between each pair of variables, with the shaded area representing the confidence interval of the regression line, indicating the uncertainty of the regression model. The wider the shaded area, the lower the confidence in the model; conversely, the narrower the shaded area, the higher the confidence in the model. In certain instances, the distribution of data points appears sparse, such as the correlation of t1 with other features/targets, t2 with other features/targets, and curing temperature with other features/targets. This data imbalance can pose challenges in model development. Figure 4 displays the Pearson correlation matrix of the precursor activator, alkali activator, process parameters, and compressive strength (target feature). Further to the moderately positive/negative correlation observed between BFS and WG/NaOH/t2, FA and NaOH, WG and NaOH/t2, NaOH and t2, and t1 and t2, other variables do not show significant correlations. These results agree with results reported in the literature [55].

### 3.2. ML Model Performance

**Figure 5:**
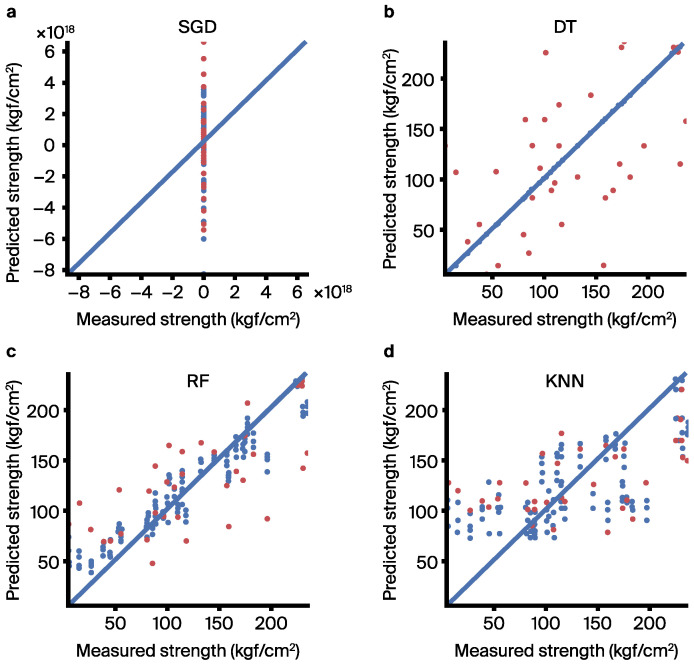
Correlation scatter plots of composition transformation dataset obtained for the ML models employed. Blue color: predicted strength (5-fold cross-validation); (**a**) SGD, (**b**) DT, (**c**) RF, (**d**) K-NN. Red color: actual strength.

**Figure 6:**
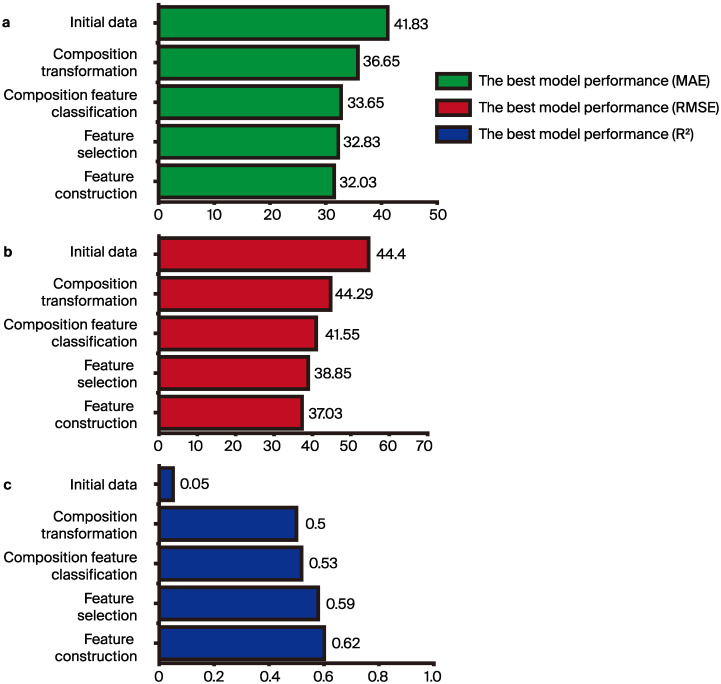
Bar chart of the model performance on each dataset. (**a**) MAE, (**b**) RMSE, and (**c**) *R*^2^.

Figure S1 and Table S4 present correlation scatter plots for each dataset used in this study that were obtained from the employed ML models, specifically SGD [49], DT [50], RF [56], and K-NN [57]. All these ML algorithms were implemented using the Python library Scikit-learn [58]. These results were obtained through fivefold cross-validation, with model performance evaluated using metrics i.e., MAE, RMSE, and *R*^2^, which are concisely summarized in Tables S4–S8. The correlation scatter plots of the initial dataset are depicted in Figure S1, accompanied by statistical error metrics provided in Table S4. The best model performance (i.e., MAE (41.83 MPa), RMSE (54.40 MPa), *R*^2^ (0.05)) for the initial dataset was achieved by K-NN model. The poor performance of the SGD model is attributed to its suitability for modeling larger datasets, whereas the initial dataset only consisted of 42 entries, resulting in suboptimal performance for SGD. Similarly, Figure 5 shows the correlation scatter plots of the composition transformation dataset, with corresponding statistical error metrics shown in Table S5. Here, the RF model demonstrates the best performance (i.e., MAE (36.65 MPa), RMSE (44.29 MPa), and *R*^2^ (0.50)). Figure S2 illustrates the correlation scatter plots of the composition feature classification dataset, and Table S6 presents the statistical error metrics. Once again, the RF model exhibits the highest performance (i.e., MAE (33.65 MPa), RMSE (41.55 MPa), and *R*^2^ (0.53)). The correlation scatter plots of the feature selection dataset are displayed in Figure S3, along with statistical error metrics in Table S7. In this case, the RF model also yields the best performance (i.e., MAE (32.83 MPa), RMSE (38.85 MPa), and *R*^2^ (0.59)). Additionally, Figure S4 presents the correlation scatter plots of the feature construction dataset, with statistical error metrics provided in Table S8. The RF model performs the best in this scenario as well. Figure 6 shows the performance of the established models in terms of the values of MAE, RMSE, and *R*^2^, respectively, using datasets processed through different data processing methods. Figure 6a–c illustrates the model evaluation results using the MAE, RMSE, and *R*^2^ metrics, respectively. Regardless of the metric used for model evaluation, the results consistently indicate a significant improvement in accuracy after the initial dataset undergoes composition transformation. This suggests that the accuracy of the models is indeed influenced by the different precursor activators and alkali activators (composition differences), aligning with the theoretical understanding that CS of AAMs is affected by the composition of precursor activators and alkali activators. Furthermore, the results show a notable improvement in accuracy after the composition transformation dataset is subjected to composition feature classification. Because the composition fields of precursor activators and alkali activators have different chemical reactions that affect AAM compressive strength [59], separating the composition fields of precursor activators and alkali activators facilitates the identification of correlations between the AAM compressive strength of the composition of precursor activators and alkali activators. Moreover, after performing feature selection on the composition feature classification dataset, there is a significant increase in model accuracy. Feature selection involved filtering out major composition components and treating minor components as impurities (other columns), and the results show that minor composition components have negligible effects on CS of AAMs. Additionally, adding six chemical indicator feature factors effectively enhances model accuracy, with these six features strongly correlated with CS (e.g., liquid binder ratio, molarity). The modeling results obtained after the aforementioned data processing demonstrate that ML models, combined with knowledge from the chemical domain (e.g., composition transformation, composition feature classification), can effectively improve model accuracy.

### 3.3. Prediction and Experimental Results

**Figure 7:**
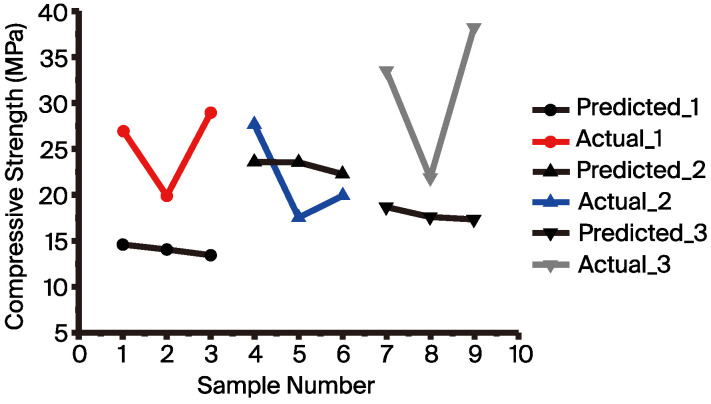
Distribution of the compressive strength of the recommended recipes.

**Figure 8:**
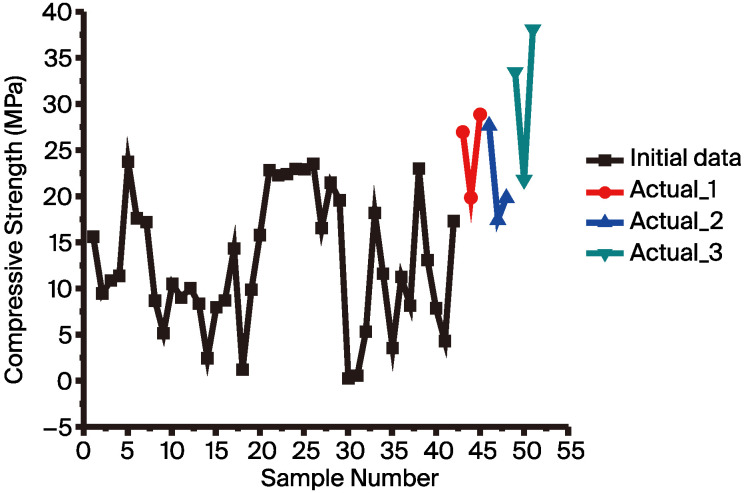
Distribution of the initial data (black), proposal 1 (red), proposal 2 (blue), and proposal 3 (gray).

To verify this result, the established model from the feature construction dataset was utilized to recommend recipes. The model utilized a dataset consisting of 42 data points. The recipes selected for experimental validation were the top three with the highest predicted compressive strength recommended by the ML model. Figure 7 shows the comparison between the predicted results and the actual experimental values for these three recipes (samples 1 to 3). The predicted compressive strengths for these recipes were 14.58 MPa, 14.04 MPa, and 13.41 MPa, respectively, while the actual compressive strengths were 26.99 MPa (SD = 0.752), 19.9 MPa (SD = 0.684), and 28.91 MPa (SD = 1.953), respectively. Although there is a significant difference between the predicted and experimentally verified compressive strength, the experimental results show that the AAMs formulation recommended by the model achieved a compressive strength of 28.91 MPa. This indicates that the model has indeed learned the formulation features associated with high compressive strength AAMs. However, because the initial data had an average compressive strength of 11.85 MPa (refer to Table 3), predicting high compressive strength recipes constitutes extrapolation for the model, leading to a large discrepancy between the predicted and actual values. In the future, continuously adding high compressive strength data can improve the model’s accuracy. Notably, the actual compressive strengths of two of the recipes were higher than the highest compressive strength of 23.84 MPa (SD = 1.222) from the initial 42 data points. Although the recipes recommended by this model did not reach the target compressive strength of 30 MPa, the literature suggests that adding new data can improve the model’s predictive ability [60]. Additionally, this study also employed the “active learning“ method from the literature, which is a cyclic optimization approach where the model recommends recipes, experiments produce new data, and this new data is then added to the model training to enhance its predictive ability. Therefore, we attempted to add three experimental recipes (Figure 7, samples 1 to 3) that obtained values close to 30 MPa, with the expectation of enhancing the model’s capability to recommend recipes. These data (45 data points) were incorporated into the training of the ML model, aiming to find recipes that achieved the target compressive strength. Samples 4 to 6 in Figure 7 show the experimental validation results when repeating the above-mentioned process. The predicted compressive strengths for these recipes were 23.64 MPa, 23.64 MPa, and 22.32 MPa, respectively, while the actual compressive strengths predicted by the ML for these recipes were 27.76 MPa (SD = 1.708), 17.58 MPa (SD = 1.964), and 19.96 MPa (SD = 1.791), respectively, meaning that we were unable to achieve the target strength. Therefore, recipes continued to be recommended in the same manner as shown in samples 7 to 9 in Figure 7. The predicted compressive strengths for these recipes were 18.63 MPa, 17.52 MPa, and 17.25 MPa, respectively, while the actual compressive strengths of these recipes were 33.42 MPa (SD = 2.283), 21.79 MPa (SD = 0.545), and 38.06 MPa (SD = 1.722), respectively. With this model established using 48 data points, the recommended recipes ultimately achieved the target compressive strength of 30 MPa. From Figure 8, it can be seen that the initial data obtained from the initial experiments (42 samples) showed a maximum compressive strength of 23.84 MPa. After two iterations of modeling (48 samples), the recommended recipe achieved a compressive strength of 38.06 MPa. Therefore, it is evident that the formula recommended by this method can effectively increase the compressive strength by 14.22 MPa (a 59.65% improvement in compressive strength).

## 4. Conclusions

This study successfully demonstrates the potential of ML techniques in predicting the compressive strength of AAMs derived from industrial waste, specifically blast furnace slag (BFS), fly ash (FA), reducing slag (RS), and waste glass (WG). By addressing the inherent challenges in traditional AAM design, which relies heavily on empirical experiments, our research provides a more efficient and cost-effective approach.

Key conclusions from this study include the following:

1. Effective data processing: Rigorous data processing, including composition transformation, feature classification, feature selection, and feature construction, was crucial in enhancing model accuracy. Each stage contributed to refining the dataset, allowing the ML models to better capture the relationships between material compositions and compressive strength. This approach significantly improved model accuracy (*R*^2^), increasing the *R*^2^ value by 0.57 (from 0.05 to 0.62), to accelerate the identification of optimal AAM recipes.
2. Significant experimental findings: The ML model, initially built with 42 data points, predicted compressive strengths for the top recipes that were subsequently validated experimentally. Although the initial predictions were below the target compressive strength of 30 MPa, iterative improvements, including the addition of new data, led to substantial gains. The final model, based on 48 data points, achieved a maximum compressive strength of 38.06 MPa, representing a 59.65% improvement. This iterative process demonstrated the ML model’s ability to significantly enhance the predictive accuracy and effectiveness of AAM recipes.
3. Overcoming traditional challenges: By addressing the time-consuming nature of traditional AAM design methods reliant on empirical experiments, our ML-driven approach offers an efficient and cost-effective alternative. It not only predicts material properties accurately but also optimizes mixtures, expediting the discovery of novel materials and mitigating the resource-intensive validation cycles. This ML method recommends recipes in just 5 min, revolutionizing AAM design in construction.

## Figures and Tables

**Table 1:** Application of ML approaches for property predictions.

Material Used	Features	Algorithm Used	Data Points	Prediction Properties	Ref.
FA	3	ANN	180	CS	[37]
FA, slag, cement	3	CNN	>5000	CS	[38]
FA	4	MARS	154	CS	[39]
FA, cement, agg.	9	BR, GD, DT, GB	645	CS, STS, EM, UTS, DSR	[40]
Slag, FA	5	SVR, RF, ET, GB	676	CS	[41]
RC, agg.	13	RF, SVM	526	EM	[42]
Cement, BFS, FA, agg.	8	ANN, XGB, SVM	100	CS	[43]
BFS, FA	2	GA	894	FST, UCS	[44]
BFS, FA, RS, WGs	27	SGD, DT, RF, K-NN	42	CS	This work

Notes: Aggregate: Agg.; Recycled concrete: RC; Splitting tensile: STS; Elastic modulus: EM; Ultimate tensile strain: UTS; Dry shrinkage rate: DSR; Final setting time: FST; Uniaxial compressive strength: UCS; ANN: Artificial neural network; Convolutional Neural Network: CNN; Multivariate Adaptive Regression Splines: MARS; Bidirectional Recognition: BR; Gradient descent: GD; Gradient Boosting: GB; Support Vector Machine: SVM; eXtreme Gradient Boosting: XGB; Genetic Algorithm: GA; DT: Decision tree; RF: Random Forest; Extra trees: ET; Stochastic Gradient Descent: SGD; K-nearest neighbors: K-NN.

**Table 2:** Chemical composition (wt. %) of the BFS, FA, RS, WG as determined by XRF-analysis.

Composition (%)	Raw Materials										
	BFS	FA ^a^	FA ^b^	FA ^c^	RS ^d^	RS ^e^	RS ^f^	WG ^g^	WG ^h^	WG ^i^	WG ^j^
Fe_2_O_3_	0.30	9.90	3.87	5.49	0.88	0.31	1.29	ND	0.10	0.06	0.43
Al_2_O_3_	15.00	20.60	28.46	21.36	3.30	2.19	7.68	5.80	18.70	18.50	2.48
SiO_2_	33.60	54.70	58.35	38.61	25.2	25.1	7.58	53.80	57.90	58.08	69.03
K_2_O	ND	1.50	1.19	1.24	ND	ND	0.05	2.80	ND	0.12	0.76
Na_2_O	ND	1.20	0.67	0.94	ND	ND	ND	5.80	ND	ND	12.87
CaO	ND	6.80	2.95	21.94	56.80	61.18	41.75	1.90	6.90	5.97	11.88
MgO	6.10	1.60	0.97	1.08	9.80	4.59	39.78	1.50	1.80	2.01	1.50

Notes: FA ^a–c^: These fly ashes refer to the particulate matter collected during the combustion process at different three power plants; RS ^d–f^: These reduction slags refer to the waste slag collected during the production processes at different three steel plants; WG ^g–j^: These waste glasses refer to the waste generated during the manufacturing processes of different four glass plants; ND: Not detected.

**Table 3:** Statistical characteristics of the variables in the initial dataset.

Variable	Unit	Min.	Max.	Average	Standard Deviation	Category
BFS	g	71.54	436.60	201.48	98.55	PAs
FA	g	0.00	300.00	133.31	70.87	PAs
WG	g	0.00	654.74	290.87	163.69	AAs
RS	g	0.00	321.43	130.40	77.18	AAs
NaOH	mL	129.54	500.00	266.96	103.37	AAs
t1	min	10.00	30.00	10.71	28.33	process
t2	min	5	30	28.33	5.31	process
Curing temperature	°C	25	92	29.95	14.33	process
CS	MPa	0.36	23.84	11.85	6.02	target feature

**Table 4:** Variable statistical characteristics of the composition feature classification dataset.

Variable	Min.	Max.	Average	Standard Deviation
Fe_2_O_3_-PAs	0.0009	0.0401	0.0148	0.0091
Al_2_O_3_-PAs	0.0429	0.1081	0.0752	0.0136
SiO_2_-PAs	0.0960	0.2764	0.1784	0.0365
K_2_O-PAs	ND	0.0060	0.0022	0.0013
Na_2_O-PAs	ND	0.0048	0.0017	0.0011
CaO-PAs	0.0541	0.1726	0.1277	0.0251
MgO-PAs	0.0094	0.0262	0.0198	0.0034
ZrO_2_-PAs	ND	0.0001	ND	ND
B_2_O_3_-PAs	ND	ND	ND	ND
TiO_2_-PAs	ND	0.0024	0.0003	0.0007
Bi_2_O_3_-PAs	ND	ND	ND	ND
BaO-PAs	ND	0.0002	ND	ND
SrO-PAs	ND	0.0003	ND	0.0001
Other-PAs	0.0120	0.0223	0.0176	0.0028
Fe_2_O_3_-AAs	ND	0.0038	0.0015	0.0010
Al_2_O_3_-AAs	0.0124	0.0931	0.0434	0.0275
SiO_2_-AAs	0.0969	0.3524	0.2406	0.0611
K_2_O-AAs	ND	0.0133	0.0063	0.0053
Na_2_O-AAs	ND	0.0642	0.0146	0.0140
CaO-AAs	0.0050	0.2489	0.0945	0.0618
MgO-AAs	0.0039	0.0562	0.0196	0.0118
ZrO_2_-AAs	0.0001	0.0156	0.0082	0.0063
B_2_O_3_-AAs	ND	0.0733	0.0496	0.0218
TiO_2_-AAs	ND	0.0089	0.0040	0.0033
Bi_2_O_3_-AAs	ND	0.0451	0.0168	0.0138
BaO-AAs	ND	0.0049	0.0012	0.0019
SrO-AAs	ND	0.0383	0.0041	0.0076
Other-AAs	0.0005	0.0160	0.0063	0.0037

Note: ND: Not detected.

## Data Availability

The ITRI-AAM database is not publicly available and, therefore, these data are not included in any of the shared files. Requests for the ITRI-AAM database should be directly sent to Hao-Yu Chan at HaoYuChan@itri.org.tw.

## Supplementary Materials

The following supporting information can be downloaded at https://www.mdpi.com/article/10.3390/ma17133141/s1, Table S1: Variable statistical characteristics of composition transformation dataset; Table S2. Variable statistical characteristics of feature selection dataset; Table S3 Variable statistical characteristics of additional features in Feature construction dataset; Table S4. Statistical error metrics of initial dataset obtained for employed ML models; Table S5. Statistical error metrics of composition transformation dataset obtained for employed ML models; Table S6. Statistical error metrics of composition feature classification dataset obtained for employed ML models; Table S7. Statistical error metrics of composition feature selection dataset obtained for employed ML models; Table S8. Statistical error metrics of feature construction dataset obtained for employed ML models; Figure S1. Correlation scatter plots of initial dataset obtained for employed ML models; Figure S2. Correlation scatter plots of composition feature classification dataset obtained for employed ML models; Figure S3. Correlation scatter plots of feature selection dataset obtained for employed ML models; Figure S4. Correlation scatter plots of feature construction dataset obtained for employed ML models.
