# Supplementary material for: Efficient Compressive Strength Prediction of Alkali-Activated Waste Materials Using Machine Learning
Source: Materials (Basel). 2024 Jun 27;17(13):3141. doi: 10.3390/ma17133141 (PMC11242840; doi:10.3390/ma17133141)
Supplement: Supplementary file 1 [file materials-17-03141-s001.zip › materials-3037420-supplementary.pdf]

Table S1. Variable statistical characteristics of composition transformation dataset.

Variable	Unit	Min	Max	Average	Standard Deviation	Category
Fe_2O_3	g	1.70	31.78	12.45	7.94	
Al_2O_3	g	39.60	153.71	85.93	34.63	
SiO_2	g	124.32	648.46	313.59	143.25	
K_2O	g	ND	21.14	7.05	6.30	
Na_2O	g	ND	40.22	13.17	12.66	
CaO	g	75.93	309.58	163.74	74.22	
MgO	g	12.76	57.79	29.09	13.55	PAs / AAs
ZrO_2	g	0.12	23.38	7.03	7.26	
B_2O_3	g	ND	108.69	39.97	29.52	
TiO_2	g	ND	11.13	3.50	3.48	
Bi_2O_3	g	ND	40.59	13.34	12.85	
BaO	g	ND	4.80	0.80	1.32	
SrO	g	ND	16.86	2.35	3.91	
Other	g	8.10	34.92	17.90	7.77	

Table S2. Variable statistical characteristics of feature selection dataset.

Variable	Min	Max	Average	Standard Deviation
Fe_2O_3-PAs	0.0009	0.0401	0.0148	0.0091
Al_2O_3-PAs	0.0429	0.1081	0.0752	0.0136
SiO_2-PAs	0.0960	0.2764	0.1784	0.0365
K_2O-PAs	ND	0.0060	0.0022	0.0013
Na_2O-PAs	ND	0.0048	0.0017	0.0011
CaO-PAs	0.0541	0.1726	0.1277	0.0251
MgO-PAs	0.0094	0.0262	0.0198	0.0034
Other-PAs	0.0120	0.0239	0.0180	0.0028
Fe_2O_3-AAs	ND	0.0038	0.0015	0.0010
Al_2O_3-AAs	0.0124	0.0931	0.0434	0.0275
SiO_2-AAs	0.0969	0.3524	0.2406	0.0611
K_2O-AAs	ND	0.0133	0.0063	0.0053
Na_2O-AAs	ND	0.0642	0.0146	0.0140
CaO-AAs	0.0050	0.2489	0.0945	0.0618
MgO-AAs	0.0039	0.0562	0.0196	0.0118
Other-AAs	0.0159	0.1273	0.0920	0.0321

Table S3. Variable statistical characteristics of additional features in Feature construction dataset.

Variable	Min	Max	Average	Standard Deviation
Add 1	0	0.1485	0.0287	0.0376
Add 2	0	0.4477	0.0816	0.1138
Add 3	0	0.1040	0.0255	0.0320
Add 4	0	0.2736	0.0706	0.0848
Add 5	0	0.1640	0.0542	0.0435
Add 6	0	0.4730	0.1522	0.1248

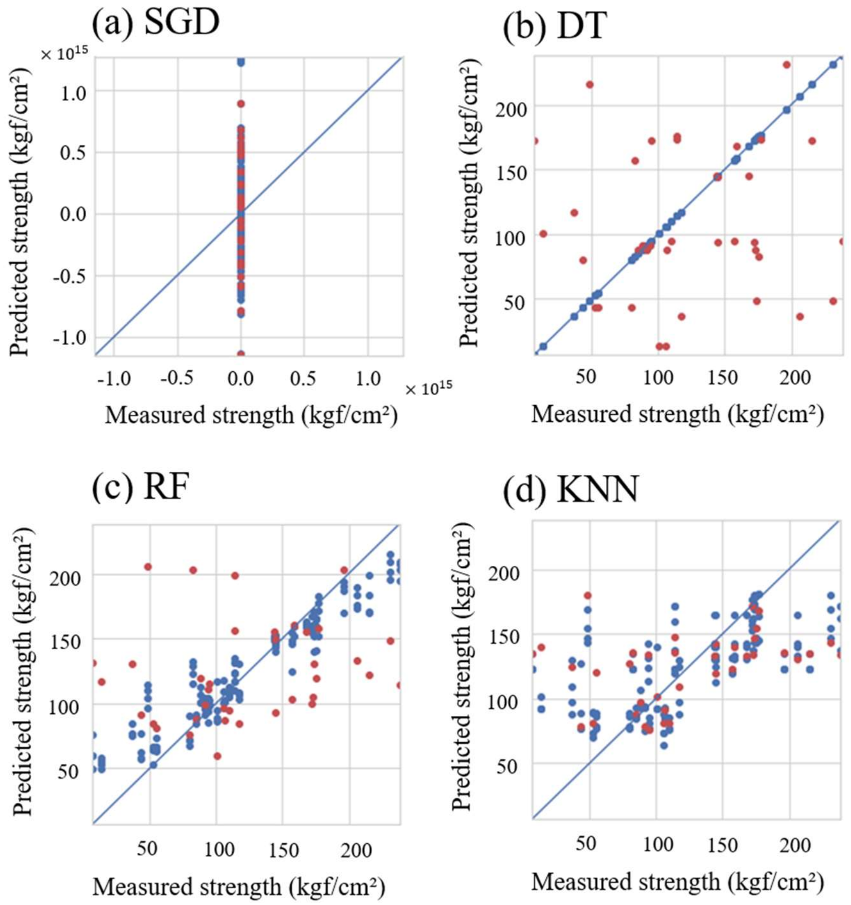

Figure S1. Correlation scatter plots of initial dataset obtained for employed ML models.

Table S4. Statistical error metrics of initial dataset obtained for employed ML models.

Metric	SGD	DT	RF	KNN
MAE	2.47×10^{14}	70.65	48.22	41.83
RMSE	2.99×10^{14}	82.00	61.82	54.40
R^2	-2.87×10^{25}	-1.17	-0.28	0.05

Table S5. Statistical error metrics of composition transformation dataset obtained for employed ML models.

Metric	SGD	DT	RF	KNN
MAE	1.74×10^{18}	46.41	36.65	45.87
RMSE	1.73×10^{18}	59.89	44.29	53.74
R^2	-2.58×10^{32}	-0.02	0.50	0.28

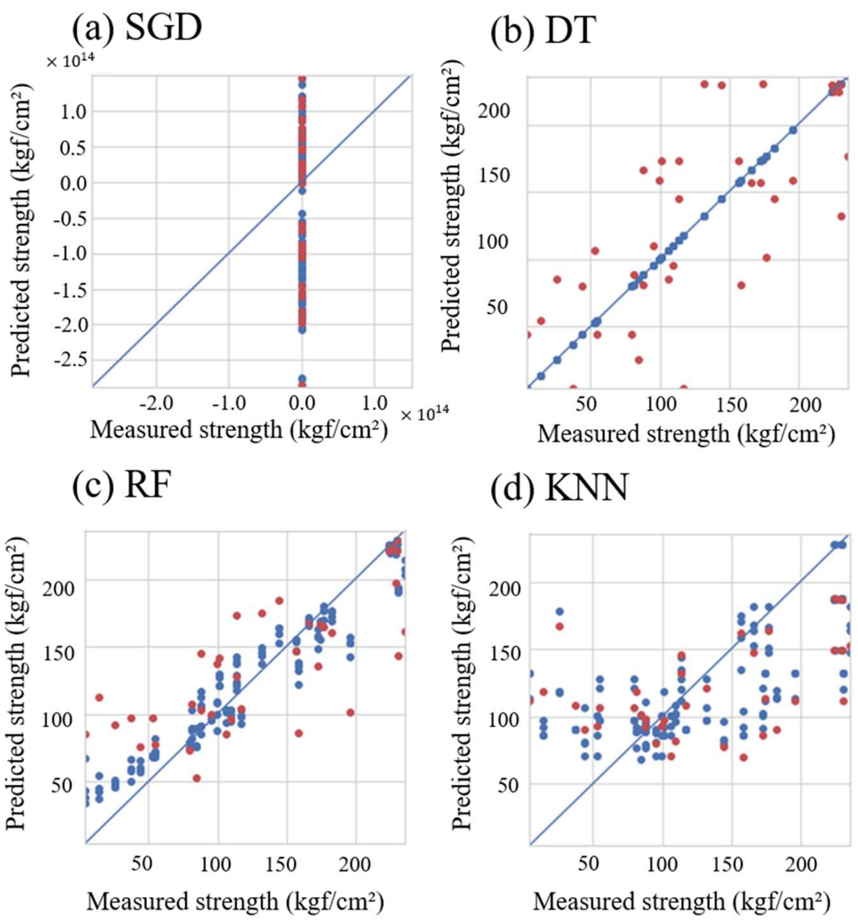

Figure S2. Correlation scatter plots of composition feature classification dataset obtained for employed ML models.

Table S6. Statistical error metrics of composition feature classification dataset obtained for employed ML models.

Metric	SGD	DT	RF	KNN
MAE	6.88×10^{13}	43.46	33.65	49.16
RMSE	1.03×10^{14}	52.46	41.55	59.56
R^2	-4.22×10^{24}	0.12	0.53	0.10

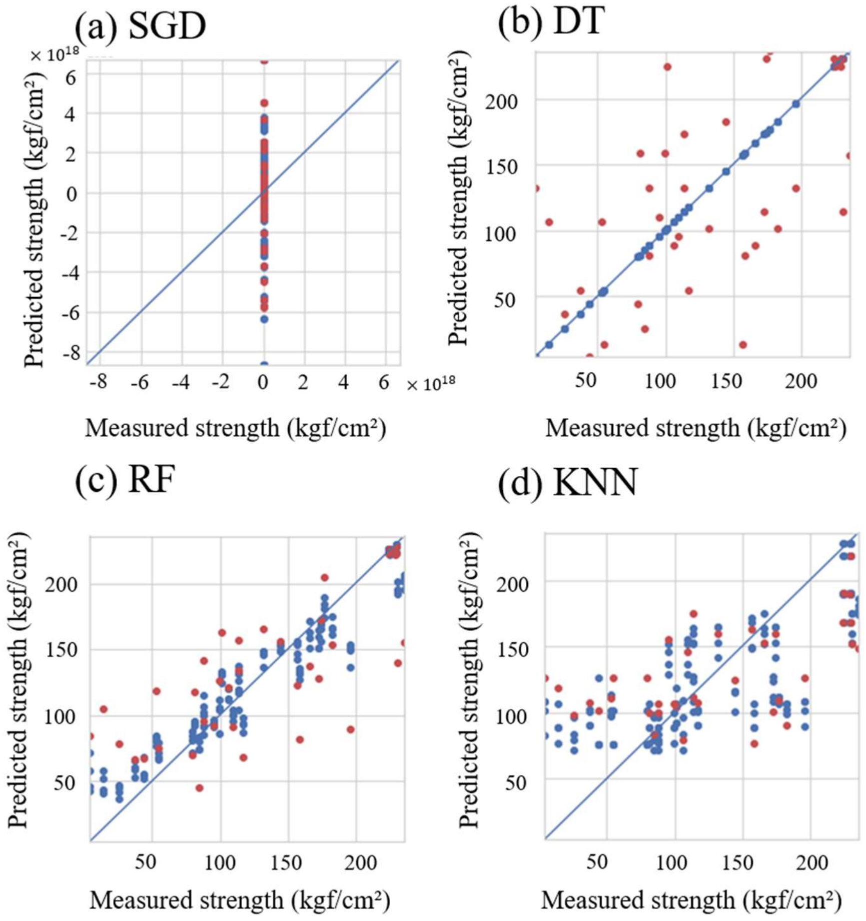

Figure S3. Correlation scatter plots of feature selection dataset obtained for employed ML models.

Table S7. Statistical error metrics of composition feature selection dataset obtained for employed ML models.

Metric	SGD	DT	RF	KNN
MAE	1.44×10^{14}	44.93	32.83	49.47
RMSE	1.09×10^{14}	52.77	38.85	59.96
R^2	-4.79×10^{24}	0.07	0.59	0.09

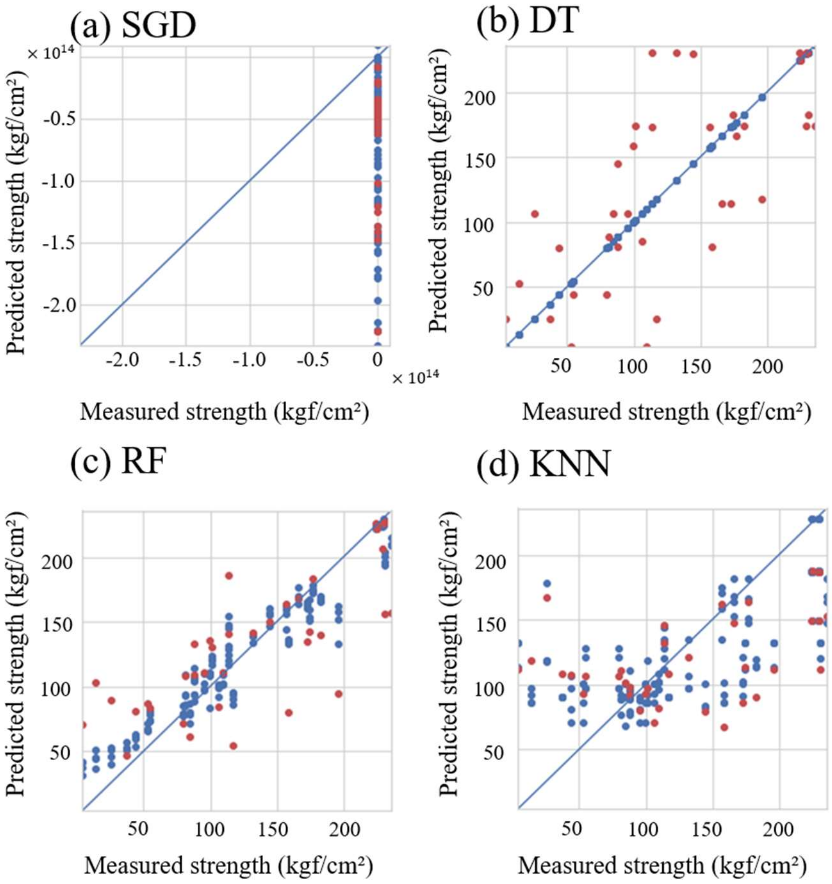

Figure S4. Correlation scatter plots of feature construction dataset obtained for employed ML models.

Table S8. Statistical error metrics of feature construction dataset obtained for employed ML models.

Metric	SGD	DT	RF	KNN
MAE	7.33×10^{13}	44.40	32.03	49.47
RMSE	4.42×10^{13}	55.30	38.93	59.96
R^2	-1.43×10^{24}	0.15	0.61	0.09
